# *MoGT2* Is Essential for Morphogenesis and Pathogenicity of Magnaporthe oryzae

**DOI:** 10.1128/mSphere.00309-19

**Published:** 2019-09-04

**Authors:** Shuzhen Deng, Wenda Sun, Lihong Dong, Guobing Cui, Yi Zhen Deng

**Affiliations:** a State Key Laboratory for Conservation and Utilization of Subtropical Agro-Bioresources, South China Agricultural University, Guangzhou, China; b Integrative Microbiology Research Centre/Guangdong Province Key Laboratory of Microbial Signals and Disease Control, South China Agricultural University, Guangzhou, China; Carnegie Mellon University

**Keywords:** *Magnaporthe oryzae*, MoGt2, pathogenesis

## Abstract

Magnaporthe oryzae causes the rice blast disease, which is one of the most serious diseases of cultivated rice worldwide. Glycosylation is an important posttranslational modification of secretory and membrane proteins in all eukaryotes, catalyzed by glycosyltransferases (GTs). In this study, we identified and characterized a type 2 glycosyltransferase, MoGt2, in *M. oryzae*. Targeted gene deletion mutants of *MoGT2* (*mogt2*Δ strains) were nonpathogenic and were impaired in vegetative growth, conidiation, and appressorium formation at hyphal tips. Moreover, *MoGT2* plays an important role in stress tolerance and hydrophobin function of *M. oryzae*. Site-directed mutagenesis analysis showed that conserved glycosyltransferase domains (DxD and QxxRW) are critical for biological functions of MoGt2. *MoGT2* deletion led to altered glycoproteins during *M. oryzae* conidiation. By liquid chromatography-tandem mass spectrometry (LC-MS/MS), we identified several candidate proteins as potential substrates of MoGt2, including several heat shock proteins, two coiled-coil domain-containing proteins, aminopeptidase 2, and nuclease domain-containing protein 1. On the other hand, we found that a conidiation-related gene, genes involved in various metabolism pathways, and genes involved in cell wall integrity and/or osmotic response were differentially regulated in the *mogt2*Δ mutant, which may potentially contribute to its condiation defects. Taken together, our results show that MoGt2 is important for infection-related morphogenesis and pathogenesis in *M. oryzae*.

**IMPORTANCE** The ascomycete fungus *Magnapothe oryzae* is the causal agent of rice blast disease, leading to severe loss in cultivated rice production worldwide. In this study, we identified a conserved type 2 glycosyltransferase named MoGt2 in *M. oryzae*. The *mogt2*Δ targeted gene deletion mutants exhibited pleiotropic defects in vegetative growth, conidiation, stress response, hyphal appressorium-mediated penetration, and pathogenicity. Furthermore, conserved glycosyltransferase domains are critical for MoGt2 function. The comparative transcriptome analysis revealed potential target genes under MoGt2 regulation in *M. oryzae* conidiation. Identification of potential glycoproteins modified by MoGt2 provided information on its regulatory mechanism of gene expression and biological functions. Overall, our study represents the first report of type 2 glycosyltransferase function in *M. oryzae* infection-related morphogenesis and pathogenesis.

## Abstract (precis)

The ascomycete fungus *Magnapothe oryzae* is the causal agent of rice blast disease, leading to severe loss in cultivated rice production worldwide. In this study, we identified a conserved type 2 glycosyltransferase named MoGt2 in *M. oryzae*. The *mogt2*Δ targeted gene deletion mutants exhibited pleiotropic defects in vegetative growth, conidiation, stress response, hyphal appressorium-mediated penetration, and pathogenicity. Furthermore, conserved glycosyltransferase domains are critical for MoGt2 function. The comparative transcriptome analysis revealed potential target genes under MoGt2 regulation in *M. oryzae* conidiation. Identification of potential glycoproteins modified by MoGt2 provided information on its regulatory mechanism of gene expression and biological functions. Overall, our study represents the first report of type 2 glycosyltransferase function in *M. oryzae* infection-related morphogenesis and pathogenesis.

## INTRODUCTION

Glycosylation is an important posttranslational modification of secretory and membrane proteins in all eukaryotes. The reaction is catalyzed by glycosyltransferases (GTs), which transfer sugar moieties from activated donor molecules to specific acceptor molecules (1). In eukaryotic cells, N- and O-glycosylation are two most common types of protein glycosylation, playing important roles during many biological processes, including protein folding, protein stability, and protein-protein interactions (2). N-glycosylation has been extensively studied in higher eukaryotes. In eukaryotic cells, a hallmark of N-glycosylation is the *en bloc* transfer of the Glc3Man9GlcNAc2 oligosaccharide to specific asparagine (Asn) residues in the Asn-Xaa-(Ser/Thr) sequence within nascent polypeptide chains. This reaction is mediated by the conserved oligosaccharyltransferase complex within the lumen of the endoplasmic reticulum (ER) (3–5). The transferred Glc3Man9GlcNAc2 oligosaccharide is then processed by the sequential action of ER glucosidases (6). Upon exit from the ER, glycoproteins are moved to the Golgi complex for further modification of N-glycans (7).

According to the stereochemistry of the substrates and reaction products, GTs can be classified into “inverting” or “retaining” enzymes (8). GTs have also been distinguished in 106 families, which are available on the continuously updated Carbohydrate-Active Enzymes (CAZy) database (http://www.cazy.org/; 9, 10). Among them, glycosyltransferase family 2 (GT2) is a large family. Recently, King et al. reported that predicted *GT2* orthologues from Zymoseptoria tritici and Fusarium graminearum are essential for fungal disease of wheat plants. In addition, *GT2* orthologues are conserved in most ascomycete filamentous fungi, but completely absent from the genomes of most ascomycete yeast species (11). Neurospora crassa *CPS-1*, a homolog of *Zymoseptoria tritici GT2*, plays an important role in vegetative growth and cell wall biogenesis (12). In Cryptococcus neoformans, the *CPS1* gene is important in pathobiology, likely serving a function in hyaluronan or its related polysaccharide synthesis, but not for protein glycosylation (13).

The ascomycete fungus Magnaporthe oryzae is the causal agent of rice blast disease, leading to severe loss in cultivated rice production worldwide (14, 15). *M. oryzae* initiates plant infection when a three-cell conidium lands on a rice leaf surface. The conidium attaches and then germinates, and the germ tube tip differentiates into a specialized infection structure called an appressorium (16). The mature appressorium generates enormous turgor (up to 8 MPa) by accumulating glycerol in the vacuole, which is used to allow a rigid penetration peg to rupture the leaf cuticle (17, 18).

Many pathogenicity-related genes in *M. oryzae* have been identified and analyzed. Such reports show that protein glycosylation is important for host infection (19–22). Chen et al. (19) found that *ALG3* (α-1,3-mannosyltransferase)-mediated N-glycosylation of the effector slp1 was essential for its activity in *M. oryzae* (19). Glycoside hydrolase *MoGLS2* deletion mutants had delayed conidial germination and showed a significant decrease in virulence and infectious growth (20). Moreover, protein *O-*mannosyltransferase members MoPmt2 and MoPmt4 are essential for *M. oryzae* morphogenesis and pathogenicity (21, 22). However, type 2 glycosyltransferases have not yet been well studied in *M. oryzae*. In this study, we identified and characterized a type 2 glycosyltransferase named MoGt2 in *M. oryzae*. We found that MoGt2 is essential for infection-related morphogenesis and pathogenesis in *M. oryzae*.

## RESULTS

### Characterization of the *M. oryzae* gene *MoGT2*.

**FIG 1:**
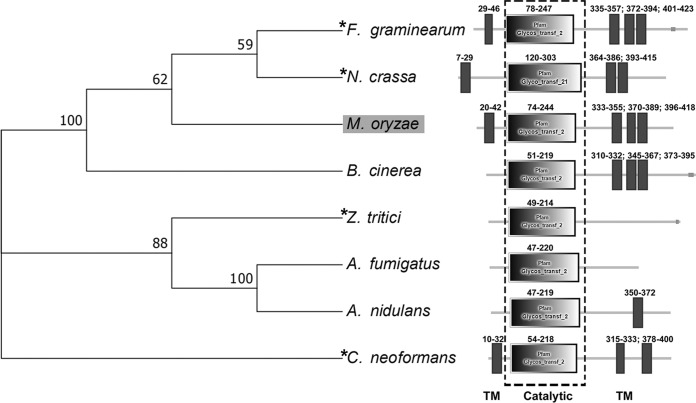
Phylogenetic tree analysis of Gt2. A neighbor-joining tree of fungal Gt2 orthologs was constructed by the MEGA version 7 program (45), including *M. oryzae* (MoGt2, MGG_01191), *F. graminearium* Gt2 (XP_011316405), N. crassa Cps-1 (XP_963800), Botrytis cinerea hypothetical protein (XP_001548088), *Z. tritici* Gt2 (XP_003857553), Aspergillus fumigatus Cps1 (XP_746682.1), Aspergillus nidulans hypothetical protein (XP_682338), and C. neoformans Cps1 (AAQ92917). The evolutionary history was inferred using the neighbor-joining method (46). The optimal tree with the sum of branch length = 1.76055829 is shown. The percentages of replicate trees in which the associated taxa clustered together in the bootstrap test (1,000 replicates) are shown next to the branches (47). The evolutionary distances were computed using the *p*-distance method (48) and are in units of the number of amino acid differences per site (labeled at the nodes). The rate variation among sites was modeled with a gamma distribution (shape parameter = 1.2). All positions with less than 50% site coverage were eliminated. The position of MoGt2 in the phylogenetic tree is indicated by gray highlighting. Asterisks denote the fungal Gt2 or Cps1 proteins characterized in pathogenic fungi (11–13). Domain annotation was performed using the SMART website (http://smart.embl-heidelberg.de/). The amino acid residue number of the annotated domains is indicated. TM, transmembrane region; catalytic, glycosyl transferase domain.

The *MoGT2* gene (MGG_01191) was identified as a 1,907-bp sequence containing 3 introns and encoding a polypeptide of 483 amino acids. Four transmembrane domains and a catalytic domain are predicted in the MoGt2 protein (Fig. 1). Phylogenetic analysis revealed that Gt2 proteins were well conserved in filamentous fungi (Fig. 1), including the orthologues in F. graminearum, N. crassa, *Z. tritici*, and C. neoformans that have been reported to play a function in fungal pathogenicity (11–13). In contrast, no orthologues have been identified in yeasts, including Saccharomyces cerevisiae, Schizosaccharomyces pombe, or the yeast-like human-pathogenic *Candida* species.

To investigate biological functions of *MoGT2*, targeted deletion of *MoGT2* was carried out with gene replacement vector pKO-GT2. Southern blot analysis confirmed that the correct gene replacement events had taken place in *mogt2*Δ targeted gene deletion mutants (*mogt2*Δ-1, -2, -4, -15, -20, -28, and -39 strains) (see Fig. S1A and B in the supplemental material). The *mogt2*Δ-28 and *mogt2*Δ-39 strains were further confirmed by real-time PCR (RT-PCR) (Fig. S1C) and selected for phenotypic analysis. To confirm the phenotypic defects of *mogt2*Δ mutants resulted from the deletion of *MoGT2*, we complemented the *mogt2*Δ-39 strain with a *MoGT2-GFP* fusion gene (C-terminal green fluorescent protein [GFP] tagging vector), and one complemented (*MoGT2-com*) strain was confirmed by RT-PCR (Fig. S1C).

10.1128/mSphere.00309-19.1 FIG S1 Targeted gene replacement of *MoGT2* and genetic complementation. Download FIG S1, TIF file, 1.9 MB. Copyright © 2019 Deng et al. 2019 Deng et al. This content is distributed under the terms of the Creative Commons Attribution 4.0 International license.

Although in the *MoGT2-com* strain, a green fluorescent protein (GFP) was tagged at the C terminus of MoGt2 protein, we were unable to observe the subcellular localization of the MoGt2-GFP fusion protein (data not shown). We performed immunoblotting with this *MoGT2-GFP* complemented *mogt2*Δ strain, using anti-GFP antibody. An 83-kDa band of the expected size of MoGt2-GFP fusion protein was detected (Fig. S1D). This confirmed that a MoGt2-GFP fusion protein was successfully expressed in the complemented strains. Meanwhile, abundant GFP peptide (of 27 kDa) was also detected (Fig. S1D), indicating that a cleavage occurred between MoGt2 and GFP. As such, the genetic complementation strain could not be used to visualize subcellular localization of MoGt2, but could still be used for assessing MoGt2 function in *M. oryzae* growth, asexual development, and pathogenicity as described in the following sections.

### *MoGT2* is necessary for vegetative growth.

**FIG 2:**
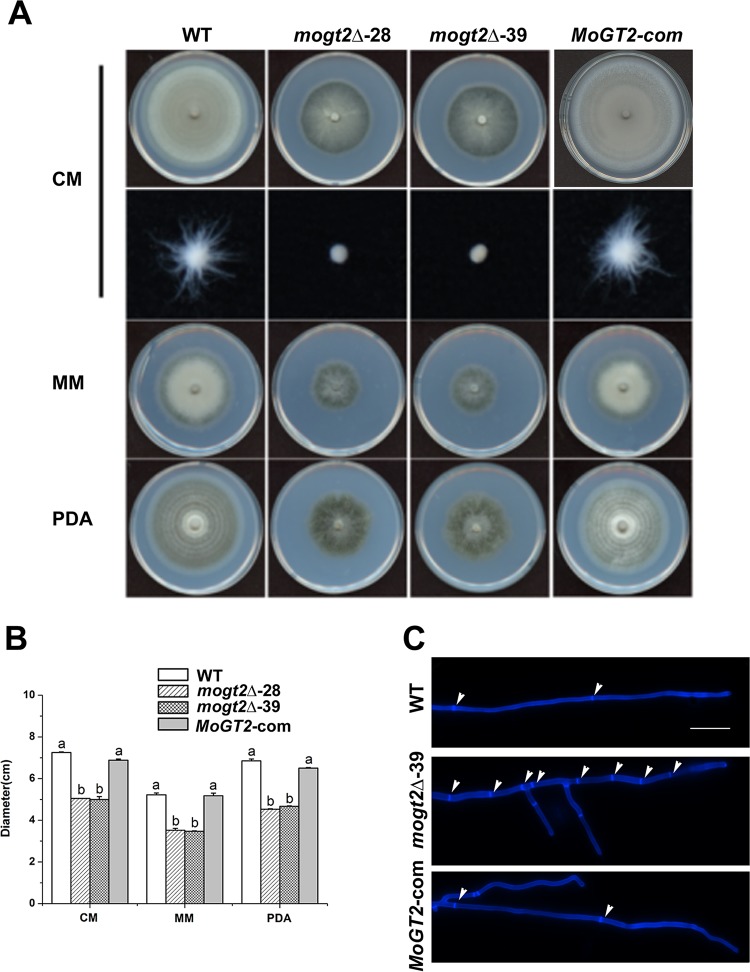
*MoGT2* is necessary for vegetative growth. (A) Colony morphology of the wild-type strain (WT), two *mogt2*Δ null mutants (*mogt2*Δ-28 and -39), and the complemented strain (*MoGT2*-*com*) grown on CM, MM, or PDA medium at 25°C. Photographs were taken at 10 days postinoculation. The second panel shows mycelial fluff of different strains, formed in liquid CM after growth at 28°C for 2 days. (B) Bar chart showing the colony diameters of the strains grown on CM for 10 days. Means and standard deviations were calculated based on three independent experiments (*n* ≥ 10). The letters a and b above the bars indicate significant differences (*P* < 0.05). (C) Hyphae of the WT, *mogt2*Δ-39, and *MoGT2-com* strains were stained with calcofluor white (18909; Sigma-Aldrich). White arrowheads point to the septa. Size bars = 20 μm.

To investigate the role of *MoGT2* in mycelial growth, we tested the growth rate of mycelia from each strain and found that *mogt2*Δ mutants grew slower than the wild-type or complemented strains, when cultured on CM (complete medium), MM (minimal medium) or PDA (potato dextrose agar) (Fig. 2A and B). When grown in liquid CM for 2 days, the *mogt2*Δ mutants formed small compact mycelial masses, in contrast to the bigger sparse mycelium formed by the wild-type (WT) or complemented (*MoGT2-com*) strain (Fig. 2A). By calcofluor white (CFW) staining, we found that the *mogt2*Δ vegetative hyphae contained more septa and the distance between two septa appeared shorter than those in the WT or *MoGT2-com* strain (Fig. 2C). These results indicated that *MoGT2* is required for proper vegetative growth in *M. oryzae*.

### *MoGT2* is essential for asexual sporulation.

**FIG 3:**
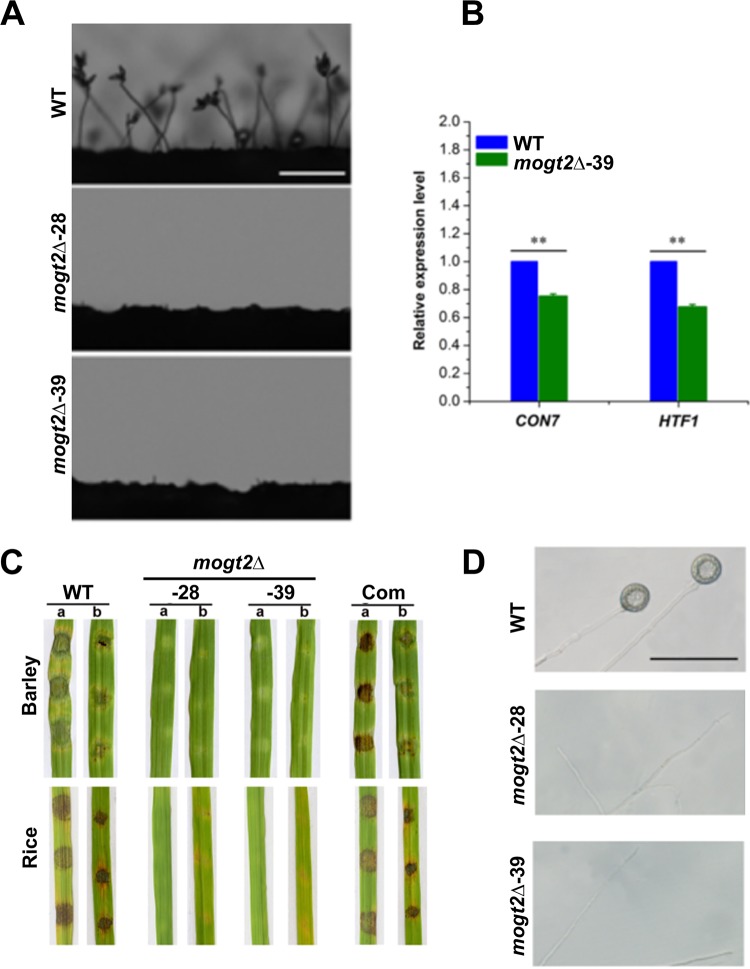
*MoGT2* is essential for *M. oryzae* conidiation and pathogenicity. (A) Microscopic observation of conidial development. Conidiophores were not observed in the *mogt2*Δ mutants, while the wild type (WT) formed normal conidiophores and conidia. Bars = 100 μm. (B) Expression levels of conidiation-related genes assessed by qRT-PCR. Means and standard deviations were calculated based on three independent experiments, each of which contains three technical replicates. Statistical difference is indicated by asterisks (*P* < 0.01). (C) Barley or rice explants were inoculated with the mycelial plugs of the strains. Photos were taken 7 days postinoculation. Com, complemented strain; a, intact leaf; b, abraded leaf. (D) Mycelium fragments of the WT or *mogt2*Δ mutant strains were placed on hydrophobic GelBond film surfaces to induce appressorium-like structure (ALS) formation. No appressorium-like structures were observed at the tip of *mogt2*Δ hyphae. Size bar = 50 μm.

Asexual spores play an essential role in the disease cycle of *M. oryzae* (14). To assess the role of *MoGT2* in asexual sporulation, we observed conidiophore differentiation and conidium production. No conidia or typical conidiophores were observed in the *mogt2*Δ mutants, while the wild-type strain formed normal conidia and conidiophores (Fig. 3A). The ability to form asexual spores was further evaluated by carefully washing the surface of different strains cultured for 10 days (16-h light/8-h dark cycle). No conidia were harvested from the *mogt2*Δ mutants, whereas the wild-type strain produced (29.6 ± 1.96) × 10^6^ spores per plate, and the complementation strain produced (24.73 ± 1.57) × 10^6^ spores per plate (*P* > 0.05, WT versus *MoGT2-com* strain). Furthermore, we tried different media for inducing conidiation, including PA (prune agar) and CM, as well as starvation conditions. Neither of them could stimulate conidiation in the *mogt2*Δ mutant. We also tried to scrape off aerial hyphae and incubate further under humid conditions, which also failed to induce conidiation in the *mogt2*Δ mutant.

Next we perform quantitative real-time PCR (qRT-PCR) analysis to check the expression levels of conidiation-related genes *CON7* and *HTF1* and found significantly reduction of these two genes in the *mogt2*Δ mutant (Fig. 3B), indicating that *MoGT2* may regulate *M. oryzae* asexual sporulation through (maybe indirectly) regulation of these conidiation-related genes’ expression. Overall, we conclude that MoGt2 is essential for *M. oryzae* conidiation.

### *MoGT2* is essential for pathogenicity and appressorium-like structure formation from mycelia.

To determine the role of *MoGT2* in plant infection, we performed infection assays with leaf explants. Since the *mogt2*Δ mutants were unable to produce conidia, we used mycelial plugs of these strains for inoculation on the surface of 7-day-old barley or 2-week-old rice leaves. After 5 days, the wild-type strain caused typical rice blast lesions on both intact and abraded leaves, while the *mogt2*Δ mutants were nonpathogenic (Fig. 3C). When inoculated on abraded barley or rice leaves, the *mogt2*Δ mutants were still unable to cause disease symptoms, suggesting that *in planta* growth was also impaired (Fig. 3C). The loss of pathogenicity was fully restored in the complementation strain (Fig. 3C, Com). The *mogt2*Δ mycelia were unable to cause disease lesion as the WT or complementation mycelia did, suggesting that MoGt2 may play a role in host infection mediated by mycelia.

*M. oryzae* can form appressorium-like structures (ALSs) at hyphal tips to penetrate plant cuticles and develop invasive hyphae (23). We also harvested the mycelium of the wild type or *mogt2*Δ mutants and induced ALSs on hydrophobic GelBond film surfaces. We found that *mogt2*Δ mutants were unable to form ALS (Fig. 3D): thus, we conclude that MoGt2 is essential for ALS formation.

### *MoGT2* is involved in stress response.

**FIG 4:**
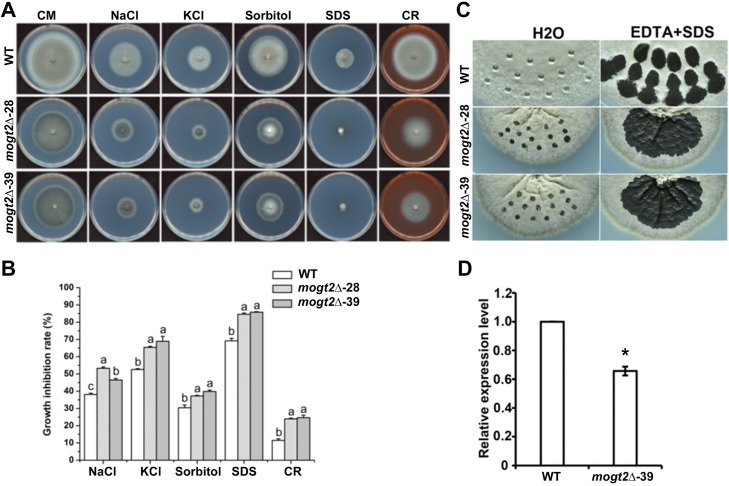
*MoGT2* is involved in stress response and hyphal hydrophobicity. (A) Colony morphology of the wild-type strain (WT) and two *mogt2*Δ mutants under various stressful conditions grown at 25°C. Photographs were taken 10 days postinoculation. (B) Calculated growth reduction rates under different stressful conditions. Growth inhibition rate (%) = [diameter (CM) − diameter (stress)]/diameter (CM). Means and standard deviations were calculated based on three independent experiments. The letters a, b, and c above the bars indicate significant differences (*P* < 0.05). (C) Droplets of water or detergent solution (0.2% SDS plus 50 mM EDTA) were placed on the surface of the wild-type (WT) or *mogt2*Δ colonies, respectively. Photographs were taken at 24 h postincubation. (D) Expression levels of hydrophobin-encoding gene *MPG1* were assessed in the WT or *mogt2*Δ strain by qRT-PCR. Means and standard deviations were calculated based on three independent experiments, each of which contains three technical replicates. Statistical difference is indicated by an asterisk (*P* < 0.01).

The fungal cell wall plays an important role in hyphal development and full virulence (24–26). We evaluated the effect of *MoGT2* disruption on stress tolerance, by assessing the growth of wild-type or mutant mycelia on CM supplemented with salt stress (0.7 M NaCl or 1.0 M KCl), the osmotic stress (1.0 M sorbitol), or cell-wall-perturbing reagents (0.01% sodium dodecyl sulfate [SDS] or 200 μg/ml Congo red [CR]). The *mogt2*Δ mutants showed significantly elevated sensitivity to various stressful conditions, as the size of mutant colonies was obviously reduced compared to those of the mutant under the untreated condition or WT colonies under the same treatment (Fig. 4A). Quantification of the growth inhibition rate based on colony diameter confirmed that the *mogt2*Δ mutants were more sensitive to these stressful conditions than the WT (Fig. 4B), suggesting that *MoGT2* plays an important role in stress tolerance in *M. oryzae*.

We noticed that after growth in liquid CM for 60 h, the hyphae of mutants became noticeably darker than those of the wild-type or complemented strains (see Fig. S2A in the supplemental material), suggesting excess melanin accumulation in the *mogt2*Δ mutants. Consistent with this, transcription levels of melanin biosynthesis genes *ALB1* and *BUF1* (27) were significantly upregulated in the *mogt2*Δ mutants compared to that of the wild-type strain (Fig. S2B), indicating that *MoGT2* is involved in regulation of melanin biosynthesis. We then used transmission electron microscopy (TEM) to examine the cell wall structure. However, no obvious differences in cell wall were observed between the wild type and the *mogt2*Δ mutants (Fig. S2C). Taken together, our results suggest that *MoGT2* plays an important role in response to various stresses.

10.1128/mSphere.00309-19.2 FIG S2 Assessment of hyphal melanization and cell wall morphology. Download FIG S2, TIF file, 2.7 MB. Copyright © 2019 Deng et al. 2019 Deng et al. This content is distributed under the terms of the Creative Commons Attribution 4.0 International license.

### *MoGT2* regulates hyphal hydrophobicity.

Surface hydrophobicity is important for pathogenicity in plant-pathogenic fungi, including the rice blast fungus (28–30). *MPG1* mutants showing an “easily wettable” phenotype, due to loss of hydrophobin production, and displayed defects in appressorium formation and disease symptom development (28). We observed that the colonies of the *mogt2*Δ mutants were morphologically distinct from the wild-type strain and failed to form appressorium; therefore, we intended to check the hydrophobicity of the *mogt2*Δ mutants. The 10-μl drops of water or detergent solutions (0.2% SDS and 50 mM EDTA) were, respectively, placed on the surface of the wild-type or *mogt2*Δ strain. We found that drops of water remained intact on the surface of the wild-type colonies after 24 h of incubation. However, the hyphae on the surface of *mogt2*Δ mutants were gradually infiltrated (Fig. 4C). When treated with detergent solutions, drops of solutions immediately soaked into the surface of *mogt2*Δ mutants and rapidly expanded to the surrounding aerial hyphae compared to the wild-type strain (Fig. 4C). These results showed that *MoGT2* regulates the hydrophobicity of aerial hyphae in *M. oryzae*.

We reasoned the wettable phenotype of *mogt2*Δ mutants may be attributed to downregulation of hydrophobin gene *MPG1*. To test this idea, we carried out quantitative RT-PCR (qRT-PCR) analysis and found that the expression levels of *MPG1* were significantly reduced in the *mogt2*Δ mutants (Fig. 4D), indicating that *MoGT2* is required for expression of *MPG1*.

### Conserved DxD and QxxRW motifs are required for *MoGT2* function.

**FIG 5:**
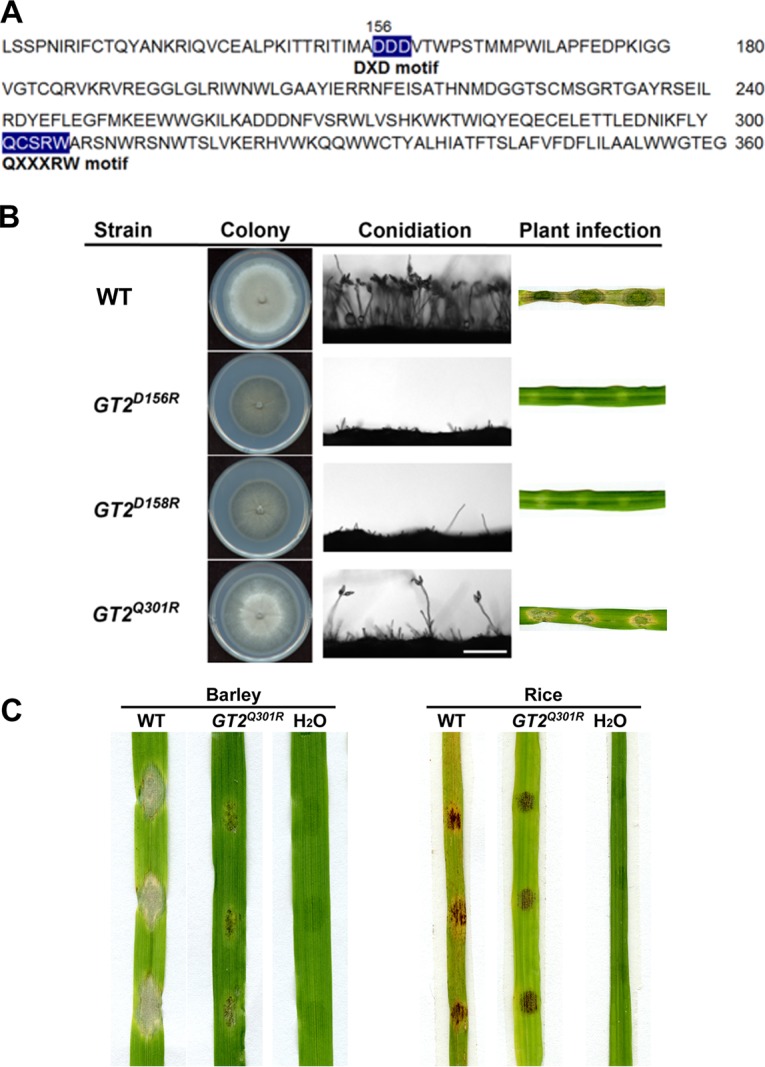
The DxD and QxxRW domains are required for the full virulence of *M. oryzae*. (A) Amino acid sequences and positions of the conserved DxD and QxxRW domains in MoGt2 protein. Two conserved domains are highlighted in blue. (B) Functional analysis of the DxD and QxxRW domains. The *GT2*^D156R^ and *GT2*^D158R^ mutants showed similar phenotypes to the *mogt2*Δ mutant; however, the reintroduction of *GT2*^Q301R^ could partially restore conidiation of the *mogt2*Δ mutant. (C) Assessment of pathogenicity of the *GT2*^Q301R^ mutant by infection assay using a conidial suspension inoculated on barley or leaf explants. The inoculum for each droplet was 2,000 conidia. Photos were taken 7 days postinoculation.

It has been reported that a number of type 2 glycosyltransferases contain conserved DxD and QxxRW motifs, which are located in nucleotide-binding and acceptor-binding domains, respectively (31–33). The DxD motif is involved in Rib and Mn phosphate coordination. Sequence analysis revealed that these two motifs are also present in MoGt2 (Fig. 5A). To study the function of these motifs, we generated point mutation constructs pGt2^D156R^, pGt2^D158R^, and pGt2^Q301R^ and introduced them into the *mogt2*Δ*-*39 mutant, respectively. The resulting *GT2*^D156R^ and *GT2*^D158R^ mutants showed similar phenotypes to the *mogt2*Δ mutant, including defective colony growth and loss of conidiation and pathogenicity (Fig. 5B). On the other hand, we observed that introduction of *GT2*^Q301R^ fragment could partially restore the vegetative growth, conidiation, and pathogenicity (Fig. 5B and C). Quantification of two *GT2*^Q301R^ strains’ conidium production was (0.20 ± 0.05) × 10^6^ and (0.30 ± 0.09) × 10^6^ conidia per plate, respectively, levels of both of which were significantly reduced compared to the wild-type strain [(29.6 ± 1.96) × 10^6^ spores per plate; *P* < 0.01]. Infection with mycelial plugs of the *GT2*^Q301R^ strain caused reduced disease lesion on the barley leaf explants (Fig. 5B). We further tested the pathogenicity of the *GT2*^Q301R^ mutant by inoculating its conidia onto barley or rice leaf explants, with the WT conidiation as a control. The results showed that the *GT2*^Q301R^ conidia were also weak in pathogenicity, compared to the wild-type conidia (Fig. 5C). Overall, these results suggested that the conserved DxD and QxxRW domains are necessary for the full function of MoGt2.

### Altered glycoproteins in *M. oryzae* conidiation due to loss of *MoGT2*.

**FIG 6:**
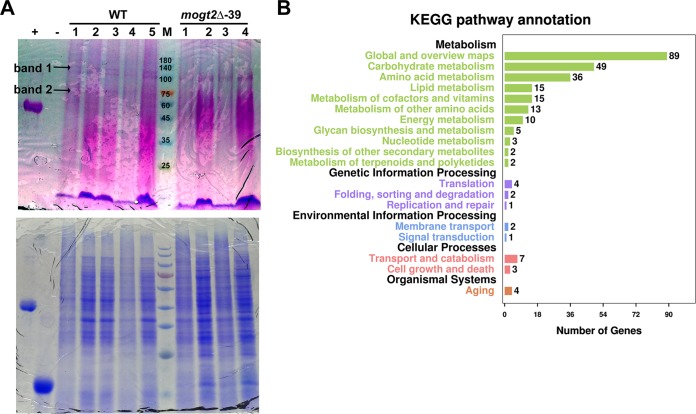
Identification of MoGt2 target protein(s) or gene(s). (A) Total protein extracts from the wild-type strain (WT) or the *mogt2*Δ mutant cultured on solid medium and exposed to light for 12 to 16 h for conidiation induction were separated by SDS-PAGE and stained with a Pierce glycoprotein staining kit (24562; Thermo Scientific) (upper panel). Coomassie blue staining (lower panel) served as a loading control. The numbers 1 to 5 for the WT samples or 1 to 4 for the *mogt2*Δ samples indicate the numbers of independent biological repeats. Arrows denote bands 1 and 2, which were present in the WT samples but absent in the mutant samples and selected for MS identification. (B) KEGG pathway enrichment of DEGs is common in three biological replicates.

To screen for the potential protein substrate(s) of the glycosyltransferase MoGt2 in *M. oryzae* during conidiation, we performed an analysis of protein glycosylation profiles with the wild-type and mutant strains. Total protein extracts from the wild-type or *mogt2*Δ mutant strain, cultured on solid medium and exposed to light for 12 to 16 h to induce conidiation, were subjected to SDS-PAGE and stained for glycoproteins. As shown in Fig. 6A, two bands, of approximately 100 to 140 kDa and 75 kDa, respectively, were present in the wild-type samples while absent in the *mogt2*Δ mutant. We cut down these two gel bands and sent them for mass spectrometry (MS) identification. In Table 1, we summarize the possible protein or proteins identified as band 1 or band 2, respectively; detailed information for peptide and protein identification is included in Data Set S1 in the supplemental material. We noticed that band 1 was most likely a coiled-coil protein-containing protein, aminopeptidase 2, or a nuclease domain-containing protein 1 (Table 1). Band 2 could also be a coiled-coil protein-containing protein (different from band 1), Hsp70, Hsp80/Hsp90, or Hsp70-like protein. A typical Hsp70 (MoSsb1) was reported critical for *M. oryzae* growth and pathogenicity and regulates the cell wall integrity (CWI) pathway governed by the mitogen-activated protein kinase (MAPK) signaling pathway (34). This Hsp70 (MoSsb1) protein was among the predicted band 2 proteins encoded by *MGG_02503*, as listed in Table 1.

**TABLE 1:** List of candidate proteins identified by mass spectrometry analysis

UniProt ID	Mol wt (kDa)	Annotation	Predicted glycosylation site(s)^*a*^	Gene ID
Band 1 (100–140 kDa)				
G4NGG4, L7IDE3, G4NGG3, L7J1X3	117–125	Hypothetical protein (coiled-coil domain containing)	N376, N1030	*MGG_04321*
L7JPS1, G4MQ02, L7HNU0	99–109	Aminopeptidase 2	N17, N47, N550	*MGG_16472*
L7HZB4, G5EHM8, L7J4A3	98	Nuclease domain-containing protein 1	N213, N345, N597	*MGG_12646*
Band 2 (∼75 kDa)				
L7HYA2, L7JL82, G4MNH8	70	Hsp70-like protein	N33, N149, N358, N416, N486	*MGG_06958*
L7JNC6, G4N0Y1, L7I4W2	75	Hypothetical protein (coiled-coil domain containing)	N268	*MGG_09571*
L7JAP8, L7HXE1, G4MKA5, A7U5U5	71–72	Glucose-regulated protein/Hsp70	No prediction	*MGG_02503*
L7JM28, L7I7P6, G4MLM8	80	Hsp80/Hsp90	N36, N71, N185, N370, N439	*MGG_06759*

a Prediction performed by NetNGlyc 1.0 Server (http://www.cbs.dtu.dk/services/NetNGlyc/).

10.1128/mSphere.00309-19.4 DATA SET S1 Detailed information of the predicted altered glycoproteins identified by mass spectrometry. Two worksheets are shown for bands 1 and 2, respectively. The header for each column is as follows (first row for protein groups and second row for peptides): Reference, reference number of protein (UniProt); PepCount, number of peptides identified in this protein; UniquePepCount, number of unique peptides in this protein; CoverPercent, percentage of unique peptides/protein; MW, molecular weight of this protein; PI, protein isoelectric point; FileScan, name of MS file for this project; Sequence, amino acid sequence for each identified peptide; MH+, theoretical molecular weight of the peptide with one H+; Diff(MH+), difference of molecular weights between detected and theoretical weights of the peptide with one H+; Charge, charge of the peptide; Rank, ranking of the peptide; Score, score of the peptide to reflect matching of the MS/MS spectrum with the library—usually threshold set as ≥20; ExpectValue, expected value by statistical analysis for matching of peptide to the library; PI, isoelectric point of the peptide; MissCleavage, trypsin cleavage at R/K may be missed; Modification, modification of the peptide would be notified—e.g., oxidation (denoted by * in the peptide) would lead to increase of molecular weight of 15.994919. Columns M to Q indicate the settings of the MASCOT software for peptide scanning. Download Data Set S1, XLSX file, 0.1 MB. Copyright © 2019 Deng et al. 2019 Deng et al. This content is distributed under the terms of the Creative Commons Attribution 4.0 International license.

### Differentially expressed genes in the *mogt2*Δ mutant during conidiation.

We also performed a transcriptome analysis between the wild-type and mutant strains under the conidiation condition. We identified 3,808 differentially expressed genes (DEGs; |log_2_| ≥ 1 and *P* ≤ 0.05), of which 1,786 overlapped in the three biological replicates (see Data Set S2 in the supplemental material). These DEGs were enriched in metabolism, genetic information processing, environmental information processing, cellular processes, and organismal systems (Fig. 6B). Particularly, a conidiation-related gene, *COS1* (35), was found significantly reduced in the *mogt2*Δ mutant compared to the WT (Data Set S2), which may support its function in *M. oryzae* conidiation. N-glycan biosynthesis was shown to be differentially regulated in the *mogt2*Δ mutant (Data Set S2), thus providing an explanation for the mutant’s altered cell wall integrity, although no morphological difference was observed by TEM (Fig. S2C). Genes involved in DNA repair or replication, protein translation, and posttranslational modification were also among the DEGs (Fig. 6B; Data Set S2). Interestingly, we noticed the autophagy pathway was enriched (Data Set S2). The MAPK pathway responsible for osmotic response was differentially regulated, consistent with the elevated sensitivity of the *mogt2*Δ mutant under osmotic or cell wall stresses (Fig. 4). We infer that MoGt2 may regulate these important metabolic and environmental response processes to fulfill its function in *M. oryzae* conidiation. On the other hand, MoGt2 may also regulate the CWI pathway and oxidative response during host infection, which is important for fungal pathogenicity (26, 36, 37).

10.1128/mSphere.00309-19.5DATA SET S2List of DEGs (*P* ≤ 0.05) and the enriched KEGG terms in the *mogt2*Δ mutant compared to the wild-type (WT) strain. The file contains six worksheets or tabs: DEGs of T-1 versus WT-1, T-2 versus WT-2, and T-3 versus WT-3 (*P* ≤ 0.05), respectively, overlapped DEGs of three sets, query to map enrichment, and map to query enrichment. T represents the *mogt2*Δ mutant, and -1, -2, or -3 represents one of the three biological repeats. For KEGG enrichment, *P* values were calculated using the formula
$P=1-\sum_{i=0}^{m-1}\frac{\left({}_{i}^{M}\right)\left({}_{n-i}^{N-M}\right)}{\left({}_{n}^{N}\right)}$ where *N* is the number of all genes with that KEGG annotation, *n* is the number of DEGs in *N*, *M* is the number of all genes annotated to specific pathways, and *m* is the number of DEGs in *M*. The calculated *P* value goes through FDR correction, taking the corrected *P* value (the *q* value) of ≤0.05 as a threshold. Download Data Set S2, XLSX file, 1.2 MB.Copyright © 2019 Deng et al.2019Deng et al.This content is distributed under the terms of the Creative Commons Attribution 4.0 International license.

## DISCUSSION

In this study, we identified and functionally characterized a predicted type 2 glycosyltransferase, MoGt2, in *M. oryzae*. MoGt2 is highly conserved among several filamentous fungi (Fig. 1) and might have conserved functions in fungal development and/or pathogenicity. Targeted deletion of *MoGT2* resulted in impairment of vegetative growth, conidiation, stress response, hyphal appressorium-mediated penetration, and pathogenicity, suggesting an important role of *MoGT2* in infection-related morphogenesis and pathogenesis in *M. oryzae*. As a member of the group 2 glycosyltransferase protein family, Gt2 contains the conserved DxD and QxxRW motifs. Our site-directed mutagenesis analysis confirmed that DxD and QxxRW motifs are critical for MoGt2 function.

Previous studies revealed that type 2 glycosyltransferase, GT2, is essential for hyphal growth in *Z. tritici* and F. graminearum (11). In *M. oryzae*, *mogt2*Δ mutants showed a similar phenotype to *Z. tritici* and F. graminearum *gt2* mutants, further confirming a conserved function of Gt2 in fungal hyphal development. Such reduced mycelial growth in the *mogt2*Δ mutants may be due to shortening of interseptal distances as visualized by CFW staining.

The *mogt2*Δ mutant failed to produce conidia under several tested culture conditions, including CM, PA (prune agar), MM, or N-deplete medium. To assess MoGt2 function in *M. oryzae* pathogenicity, we performed an infection assay using the mycelial plugs as the *mogt2*Δ mutant did not produce conidia. We found that the *mogt2*Δ mutant was unable to invade host tissue or form an appressorium-like structure from mycelia to penetrate the host cuticle. Therefore, we reasoned that the loss of virulence in the *mogt2*Δ mutant may be caused by deficiency in appressorium-like structure formation. To get a better understanding of the role of MoGT2 in infection awaits silencing of this gene only during infection and observation of pathogenicity under such a condition. In addition, we found the *mogt2*Δ mutants showed an “easily wettable” phenotype and reduction in hydrophobin gene *MPG1* transcription (Fig. 4D), which may account for impairment of conidium production and hyphal growth.

A *MoGT2-GFP* fragment was reintroduced into the *mogt2*Δ mutant and able to restore all phenotypes, indicating that the ectopically expressed MoGt2-GFP fusion protein is functional. However, we failed to detect visible GFP signal in the complementation strain under mycelial growth or conidiation or during the infection stage. The predicted topology of MoGt2 is that its C terminus is outside the plasma membrane, so we infer that the C-terminal GFP was cleaved and released to the extracellular space and therefore could not be used to assess subcellular localization of MoGt2.

Cell wall is an important structure that is responsible for maintaining cell shape and is also critical for cell expansion during growth and morphogenesis (49). In *M. oryzae*, cell wall integrity was essential for fungal pathogenesis (26, 36, 37). In this study, deletion of *MoGT2* led to increased sensitivity to distinct stresses, including the osmotic stress and cell-wall-perturbing reagents. In N. crassa, it has also been reported that *cps-1* deletion mutants are sensitive to cell wall perturbation reagents and play a critical role in cell wall biogenesis (12). However, TEM observation showed no obvious differences in cell wall ultrastructure between the wild type and *mogt2*Δ mutants (Fig. S2C).

By liquid chromatography-tandem MS (LC-MS/MS), we tried to identify two bands cut from SDS-PAGE for the glycoproteins present in the wild-type strain but absent in the mutant during conidiation. We identified two coiled-coil domain-containing proteins, several heat shock proteins, aminopeptidase 2, and nuclease domain-containing protein 1. Particularly, a typical Hsp70 protein, MoSsb1, was recently reported to be important for *M. oryzae* growth, conidiation, and pathogenicity and regulates the CWI pathway through interaction with MAPK MoMkk1. The *mossb1*Δ mutant displayed similar phenotypes to the *mogt2*Δ mutant, except that the *mossb1*Δ mutant produced conidia but of abnormal morphology (34). We infer that MoGt2 may regulate *M. oryzae* growth, pathogenicity, and CWI through glycosylation of MoSsb1, and other (unidentified) substrates may contribute to conidiation. However, we failed to predict any glycosylation residue on MoSsb1 by using the NetNGlyc 1.0 Server (Table 1). Whether MoSsb1 is actually glycosylated by MoGt2, as well as the biological relevance of such posttranslational modification, awaits further investigation. Verification of other potential substrates listed in Table 1 would also be of interest.

We also performed comparative transcriptome analysis to investigate the mechanism of MoGt2 function. A conidiation-related gene, *COS1* (35), was found significantly reduced in the *mogt2*Δ mutant compared to the WT (Data Set S2). Two conidiation-related genes, *CON7* and *HTF1*, were not among the filtered DEGs (|log_2_| ≥ 1 and *P* ≤ 0.05) but were shown by qRT-PCR analysis to be downregulated in the *mogt2*Δ mutant (Fig. 3B). The melanin biosynthesis genes *ALB1* and *BUF1* were found significantly upregulated, and the hydrophobin-encoding gene *MPG1* was downregulated, in the *mogt2*Δ mutant compared to the WT, which was consistently supported by the comparative transcriptome analysis (Data Set S2) and qRT-PCR (Fig. 4D; Fig. S2B). This confirms that the results from comparative transcriptome analysis were reliable. Functional investigation of the candidate DEGs may help further elucidate the MoGt2 functional mechanism. Nuclease domain-containing protein 1 was identified as a potential glycoprotein in the wild-type strain during conidiation, which also contains a Tudor domain that was reported present in RNA-binding proteins (38). This potential RNA-binding nuclease may account for the DEGs between the wild type and the *mogt2*Δ mutant and is likely subject to regulation through glycosylation. Three glycosylation residues (Asn in the Asn-Xaa-Ser/Thr sequon) could be predicted in this protein (Table 1). Such a hypothesis needs further verification in the future.

Overall, our study identified a type 2 glycosyltransferase, MoGt2, in *M. oryzae*, responsible for vegetative hyphal growth, conidiation, pathogenicity, and CWI. In the present study, we did not perform an *in vitro* experiment to test the glycosyltransferase activity of MoGt2, nor did we confirm the biological function of candidate glycoproteins in *M. oryzae* conidiation and their relationship with MoGt2. Future investigation of these aspects will further reveal the cellular and molecular mechanisms of MoGt2 function in fungal development and pathogenicity.

## MATERIALS AND METHODS

### Strains and culture conditions.

Magnaporthe oryzae strain Guy11 was used as the wild-type strain. The wild-type strains and corresponding transformants generated in this study were grown on CM at 25°C for 10 days. For the stress sensitivity test, the mycelial plugs of each strain were cultured on CM plates, to which were added 0.7 M NaCl, 1 M KCl, 1 M sorbitol, 0.01% SDS, and 0.2 mg/ml Congo red (CR), respectively. The diameters of the colonies were recorded 10 days after inoculation. Conidial development was assessed by harvesting conidia from the surface of 10-day-old plate cultures and determining the concentration of the resulting conidial suspension using a hemocytometer (Corning). Means and standard deviations were calculated based on three independent experiments.

### Nucleic acid manipulation, qRT-PCR, and Southern blotting.

General procedures for nucleic acid analysis followed standard protocols (39). Total RNA was extracted using PureLink RNA minikit (Invitrogen, USA) and used to synthesize first-strand cDNA using PrimeScript RT (TaKaRa). The qRT-PCR was performed on the QuantStudio 6 Flex (Applied Biosystems, USA) by using SYBR green PCR master mix (Vazyme, Nanjing, China) as per the manufacturer’s instruction. Genomic DNAs were extracted from vegetative hyphae with the cetyltrimethylammonium bromide (CTAB) protocol (40). Southern blot analysis was performed with the digoxigenin (DIG) High Prime DNA labeling and detection starter kit II (Roche, Mannheim, Germany). The primers used in this study are listed in Table S1 in the supplemental material.

10.1128/mSphere.00309-19.3 TABLE S1 Primers used in this study. Download Table S1, DOCX file, 0.1 MB. Copyright © 2019 Deng et al. 2019 Deng et al. This content is distributed under the terms of the Creative Commons Attribution 4.0 International license.

### Plasmid constructs and fungal transformants.

To construct the *GT2* gene replacement vector pKO-GT2, the 1.5-kb upstream and 1.5-kb downstream sequences of *MoGT2* were amplified with primer pairs LB F/LB R and RB F/RB R, respectively. The two flanking sequences were cloned into the pFGL821 vector to generate pKO1191.Then pKO1191 was transformed into Guy11 protoplasts to generate homologous recombinants, as previously described (28).

For *GT2* complementation, a 3.7-kb fragment, including a 1.8-kb native promoter region and 1.9-kb full length of the *MoGT2* gene, and the enhanced green fluorescent protein (eGFP) gene were amplified and then cloned into pCB1532 to create pGT2:eGFP (a C-terminal GFP tagging vector), according to the manufacturer’s instructions of the One Step cloning kit (Vazyme, Nanjing, China).

For generating the *GT2*^D156R^ mutant, site-directed mutagenesis introduced D156R R/D156R F to replace D with R (GAT to CGA) in the Gt2 domain. Fragments of 2.1 and 1.8 kb were amplified with primer pairs Gt2 up F/D156R R and D156R F/Gt2 down R, respectively. Two PCR products were cloned into pCB1532 to create pD151R with the One Step cloning kit, as described above. The resulting plasmid was transformed into the *mogt2*Δ-39 mutant to generate the *GT2*^D156R^ mutant. Similar strategies were used to generate other site-directed mutants.

### Pathogenicity assay.

Two-week-old seedlings of the rice cultivar CO39 and 7-day-old seedlings of the barley cultivar Golden Promise were used for infection assays. The mycelium plugs from 10-day-old CM cultures were placed onto the leaf surface. Wounded leaves were prepared by removing the surface cuticle by abrasion with an emery board (41). The inoculated plants were incubated in a plastic plate with full humidity at 25°C. The disease lesions were examined and photographed at 5 days postinoculation.

### Transmission electron microscope.

Mycelium cultured in liquid CM for 2 days was processed for transmission electron microscopy (TEM). TEM sample treatment was performed as described previously (42), and treated samples were observed under a transmission electron microscope (Hitachi H-7650).

### Staining assays.

For calcofluor white staining, mycelia were stained with 10 μg/ml CFW (18909; Sigma-Aldrich) for 10 min in the dark and then washed twice with phosphate-buffered saline (PBS) buffer.

### Glycoprotein staining.

The wild-type and mutant strains were grown in liquid CM for 2 days before harvesting the mycelia. Total protein extract from the mycelia was subjected to SDS-PAGE and stained using a Pierce glycoprotein staining kit (24562; Thermo Scientific).

### Mass spectrometry.

The protein gel band cut from the SDS-PAGE gel was digested with trypsin for 20 h at 37°C. The digested peptides were loaded onto a reverse-phase trap column (Thermo Scientific Acclaim PepMap100, 100 μm by 2 cm; nanoViper C_18_) connected to the C_18_ reverse-phase analytical column (Thermo Scientific Easy column, 10 cm long, 75-μm inner diameter, 3-μm resin) in buffer A (0.1% formic acid) and separated with a linear gradient of buffer B (84% acetonitrile and 0.1% formic acid) at a flow rate of 300 nl/min (0 to 35% buffer B for 50 min, 35 to 100% buffer B for 5 min, hold in 100% buffer B for 5 min). Bioinformatic analysis of raw LC-MS/MS data followed the established protocol (43) and was performed by Applied Protein Technology Co., Ltd. (Shanghai, China).

### Western blot analysis.

Total protein was extracted from mycelium cultured in liquid CM for 2 days and separated on a 10% SDS-PAGE gel, before being transferred to polyvinylidene difluoride (PVDF) membrane. Detection of GFP fusion proteins was carried out using anti-GFP antibody (A6455, rabbit, 1:5,000; Invitrogen Molecular Probes). The SuperSignal West Pico chemiluminescent kit (34580; Thermo Scientific) was used for signal detection.

### RNA-seq and transcriptome analysis.

High-throughput RNA sequencing (RNA-seq) and transcriptome analysis were performed by Gene Denovo Co. (Guangzhou, China) using the reported protocols (43). Short reads were mapped to the complete genome of *M. oryzae* (https://www.ncbi.nlm.nih.gov/genome/?term=magnaporthe+oryzae) using Tophat (44). Genes with a fold change of 2 and a false-discovery rate (FDR) of 0.05 in a comparison of significant differentially expressed genes (DEGs) were subjected to enrichment analysis of Gene Ontology (GO) functions and KEGG pathways following our established protocol (43).
