# Supplementary figures and images for: MoGT2 Is Essential for Morphogenesis and Pathogenicity of Magnaporthe oryzae
Source: mSphere. 2019 Sep 4;4(5):e00309-19. doi: 10.1128/mSphere.00309-19 (PMC6731526; doi:10.1128/mSphere.00309-19)

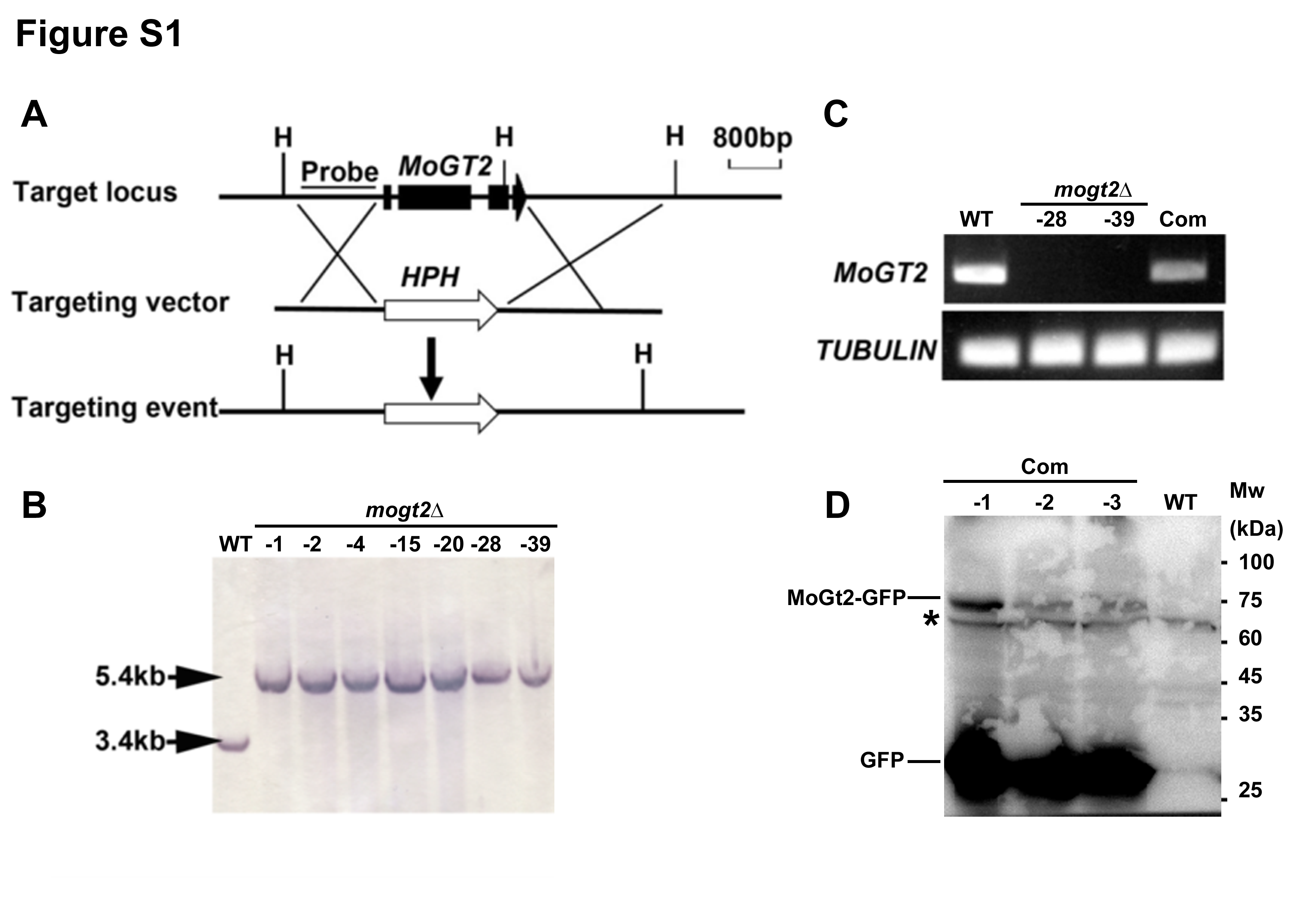

Supplement: FIG S1 [file mSphere.00309-19-sf001.tif]

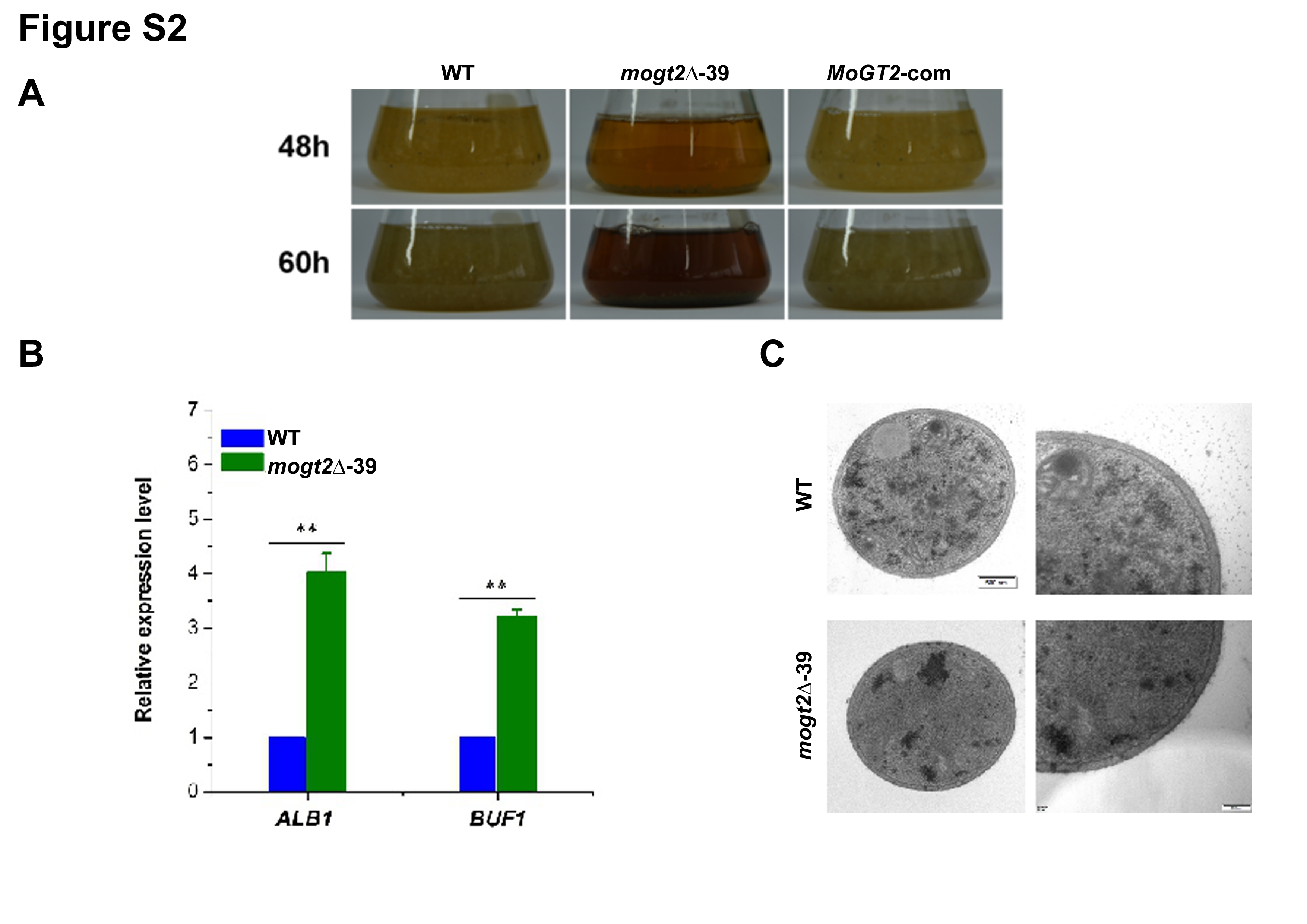

Supplement: FIG S2 [file mSphere.00309-19-sf002.tif]
