# Supplementary material for: MoGT2 Is Essential for Morphogenesis and Pathogenicity of Magnaporthe oryzae
Source: mSphere. 2019 Sep 4;4(5):e00309-19. doi: 10.1128/mSphere.00309-19 (PMC6731526; doi:10.1128/mSphere.00309-19)
Supplement: TABLE S1 [file mSphere.00309-19-st001.docx]

**Table S1 Primers used in this study.**

| **Name** | **Sequence (5’-3’)** | **Purpose** |
| --- | --- | --- |
| LB F1 | CATGGTACCCGTCTCGCGGTAGATATTTG | *MoGT2*  deletion |
| LB R1 | TACGGATCCCAGAAATGCAGTGGATTCTTC |  |
| RB F1 | CATCTGCAGTAACGCCTTATGGATTGAACG |  |
| RB R1 | ACTAAGCTTCGTCTGCTCCTTGAATCTAT |  |
| In F | ACGACGTTACCTGGCCATCG | Verification of *MoGT2*  deletion |
| In R | GACATGACGCTCCTTGACCA |  |
| Check F | CTACCTAGGACTGTGCTGTA |  |
| Hph R | GGCTGATCTGACCAGTTGCC |  |
| Tublin F1 | GTTCACCTTCAGACCGG |  |
| Tublin R1 | GAGATCGACGAGGACAG |  |
| hb F | GGATCCCCCGGGCTGCAGGAATTCCTACCTAGGACTGTGCTGTA | Generation of  *MoGT2*  Complementation |
| hb R | CAGCTCCTCGCCCTTGCTCACCATCAGGTGCTCCCATTCCTCTT |  |
| Gt2 up F | TCCCCCGGGCTGCAGGAATTCCTACCTAGGACTGTGCTGTA | *MoGT2* site - directed  mutagenesis |
| Gt2 down R | GATAAGCTTGATATCGAATTCGAAACCAAAATCACCCATTCC |  |
| D156R R | TCGGGCCATAATAGTGATGC |  |
| D156R F | GCATCACTATTATGGCCCGAGACGACGTTACCTGGCCATCG |  |
| D158R R | TCGGTCATCGGCCATAATAG |  |
| D158R F | CTATTATGGCCGATGACCGAGTTACCTGGCCATCGACCAT |  |
| D301R R | TCGGTAGAGGAACTTGATGT |  |
| D301R F | ACATCAAGTTCCTCTACCGATGCTCGAGGTGGGCCAGGAG |  |
| βtub-q-F | CATACGGTGACCTGAACTAC | qRT-PCR  analysis |
| βtub-q-R | CCATGAAGAAGTGCAGACG |  |
| CON7-q-F | AGTGGCAGCAGTGGAGATC |  |
| CON7-q-R | GCGGTTGGGCATAGAGGTT |  |
| HTF1-q-F | GATGGATTCTCAGTCTCGG |  |
| HTF1-q-R | AATGACGATGGAGCCGCTTG |  |
| ALB1-q-F | TGACACCTTCCTCAACACC |  |
| ALB1-q-R | CGAGCCAGATTTAAGCAGCC |  |
| BUF1-q-F | TACAAGCACCTCGAGATTGG |  |
| BUF1-q-R | CAGTAATCTTCTTGTCGGCC |  |
| MPG1-q-F | GAAGGTCGTCTCTTGCTGCA |  |
| MPG1-q-R | GGATGTTGACCAGACCAATC |  |
| MHP1-q-F | CACCATCATCGCCACCATC |  |
| MHP1-q-R | CAGCACTGAGCAGAGCCGTA |  |
